# Triple sheath neuroendoscopic combination technique for managing complete intraventricular hemorrhage casting in patients with cerebral hemorrhage

**DOI:** 10.3389/fneur.2025.1554187

**Published:** 2025-06-04

**Authors:** Zohaib Shafiq, Long Zhou, Xi Jiang, Zhiyang Li, Ping Song, Silei Zhang, Qiang Cai

**Affiliations:** ^1^Department of Neurosurgery, Renmin Hospital of Wuhan University, Wuhan, China; ^2^Department of Neurosurgery, Hubei Provincial Hospital of Traditional Chinese Medicine, Wuhan, China; ^3^Department of Neurosurgery, Xiantao First People’s Hospital, Xiantao, China

**Keywords:** triple sheath neuroendoscopic combination technique, intraventricular hemorrhage casting, cerebral hemorrhage, midbrain aqueduct, neuroendoscopy, surgical outcomes

## Abstract

**Objective:**

To evaluate the efficacy of the triple sheath neuroendoscopic combination technique (TSNCT) compared to standard endoscopic hematoma removal and external ventricular drainage (EVD) for managing complete intraventricular hemorrhage (IVH) casting in patients with cerebral hemorrhage.

**Methods:**

A retrospective analysis was conducted on five patients with complete IVH casting treated at our institution between 2023 and 2024, including two treated with TSNCT, two with standard endoscopic hematoma removal, and one with EVD. Preoperative and postoperative imaging, intraoperative neuroendoscopic video, and clinical data were reviewed. The TSNCT involves an outer sheath for ventricular access, a middle sheath for maneuverability, and a mini sheath designed to navigate the midbrain aqueduct.

**Results:**

TSNCT enabled near-complete evacuation of hematomas, including in the midbrain aqueduct, achieving a mean hematoma clearance rate of 93.5% in the two TSNCT cases, compared to 91.7% for standard endoscopic removal and 27.2% for EVD. TSNCT cases showed greater neurological improvement [mean Glasgow Coma Scale (GCS) increase of 7 points] than standard endoscopic removal (3 points) and EVD (no improvement). TSNCT addresses high mortality associated with severe hemorrhage involving all ventricular chambers, with fewer complications in this small cohort.

**Conclusion:**

TSNCT offers a novel approach to overcome anatomical challenges in complete IVH casting, enhancing surgical precision and showing potential for improved patient outcomes. Further research with larger cohorts is needed to validate these preliminary findings and standardize its application in neurosurgical practice.

## Introduction

The concept of endoscopy dates back centuries, but practical neuroendoscopic systems only emerged in the early 1990s (1, 2). Since then, neuroendoscopy has undergone significant advancements due to technological developments and interdisciplinary influences, leading to its widespread adoption in modern neurosurgery (3). Recently, neuroendoscopy has been employed in various procedures, including intraventricular, skull base, and spinal surgeries (4).

IVH is a life-threatening condition often associated with high morbidity and mortality (5). Traditional treatments, such as EVD, have been widely used to relieve ICP and manage hydrocephalus (6). However, EVD has limitations in effectively evacuating hematomas, often leaving residual clots that obstruct cerebrospinal fluid pathways and worsen patient outcomes (7, 8).

**Figure 1:**
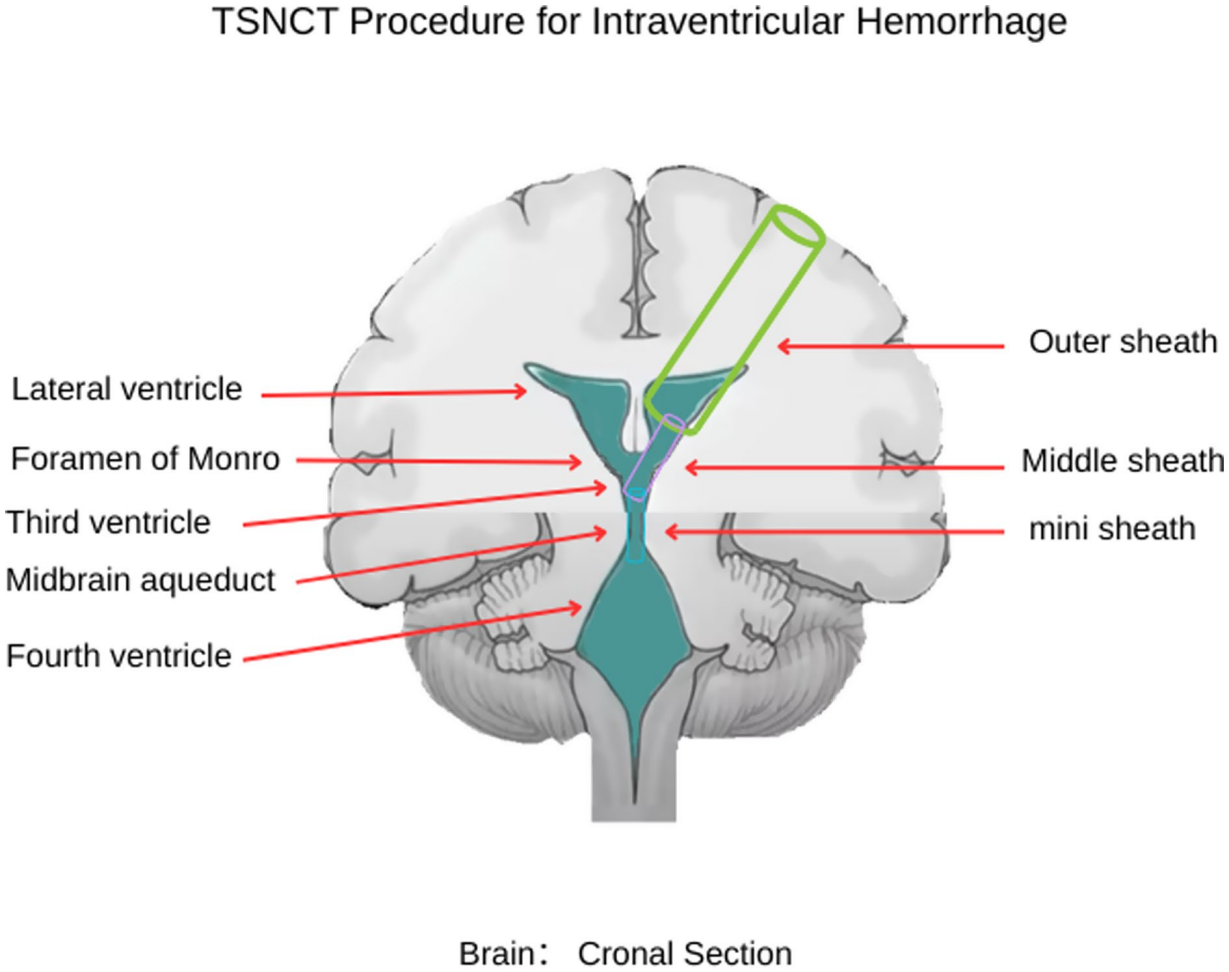
Schematic illustration of the TSNCT. The outer sheath is positioned in the lateral ventricle (→ green), the middle sheath navigates through the foramen of Monro to the third ventricle (→ pink), and the mini sheath extends through the midbrain aqueduct to access the fourth ventricle (→ blue).

To address these challenges, advanced neuroendoscopic techniques, such as single-port rigid and flexible endoscopy, have been developed (9–11). These methods use a small burr hole to insert an endoscope, enabling hematoma evacuation with tools like suction and irrigation (9). Rigid endoscopy (e.g., Karl Storz, system) offers high-resolution imaging for lateral and third ventricle access, while flexible endoscopy enhances maneuverability (10). While these techniques reduce invasiveness and provide real-time visualization (11), limited access to the fourth ventricle due to the narrow midbrain aqueduct (≈2 mm) (Figure 1) restricts outcomes in complete IVH casting (12, 13).

This article introduces the novel TSNCT, which specifically addresses the complex issues associated with managing complete intraventricular casts resulting from cerebral hemorrhage. The TSNCT comprises the following: The outer sheath provides secure access to the ventricular system. The middle sheath enhances maneuverability while protecting adjacent structures at the foramen of Monro. The mini sheath is designed for navigating the delicate midbrain aqueduct, offering unprecedented access to previously difficult areas.

As illustrated in Figure 1, TSNCT employs these sheaths to achieve comprehensive hematoma evacuation across all ventricular chambers. This pilot observational study explores the feasibility, safety, and preliminary efficacy of TSNCT in a small cohort of patients with complete IVH casting. By examining clinical presentation, imaging characteristics, surgical strategy, and initial outcomes, we aim to assess TSNCT’s potential to enhance surgical precision, minimize brain tissue damage, and improve neurological outcomes compared to existing methods, providing a foundation for future research into severe IVH management.

## Materials and methods

### Study design

This pilot observational study involves a retrospective analysis of patients diagnosed with complete intraventricular casts due to cerebral hemorrhage who were treated at our institution between 2023 and 2024. The study was approved by the ethics committee of our institution, and written informed consent was obtained from all participants or their legal representatives in accordance with the 1964 Helsinki Declaration and its amendments.

### Patient selection

A cohort of five patients (three males, two females; age range 52–70 years) was selected based on medical records and imaging data. Detailed clinical, surgical, and outcome data are presented in Table 1.

**Table 1:** Clinical, surgical, and outcome data.

No.	Gender	Age	Diagnosis	Technique	Preoperative hematoma volume (mL)	Postoperative hematoma volume (mL)	GCS at admission	GCS at discharge	Operation time (min)	Drainage tube removal time (days)	Length of hospital stay (days)	Use of urokinase
1	M	61	Complete IVH	TSNCT	76.1	3.6	4 T	14	110	3	17	No
2	F	70	Complete IVH	TSNCT	48	4.5	4 T	8 T	90	4	17	No
3	M	66	Complete IVH	Endoscopic hematoma removal	87.5	10.8	4 T	8 T	100	5	10	Yes
4	F	57	Complete IVH	Endoscopic hematoma removal	56.4	1.1	5 T	7 T	130	4	39	No
5	M	52	Complete IVH	EVD	61.3	44.6	3	3	25	5	5	Yes

“T” signifies intubated patients where the verbal component cannot be assessed.

#### Inclusion criteria

Confirmed diagnosis of complete IVH casting involving all ventricular chambers (lateral, third, and fourth ventricles) via computed tomography (CT). Treatment with TSNCT, standard endoscopic hematoma removal, or EVD.

#### Exclusion criteria

Incomplete IVH casting (not involving all ventricles). Contraindications to surgery (e.g., uncorrectable coagulopathy). Data were collected from preoperative and postoperative CT scans, intraoperative neuroendoscopic video recordings, and clinical records, including GCS assessments and complication reports.

### Interventions

#### TSNCT group (*n* = 2)

**Figure 2:**
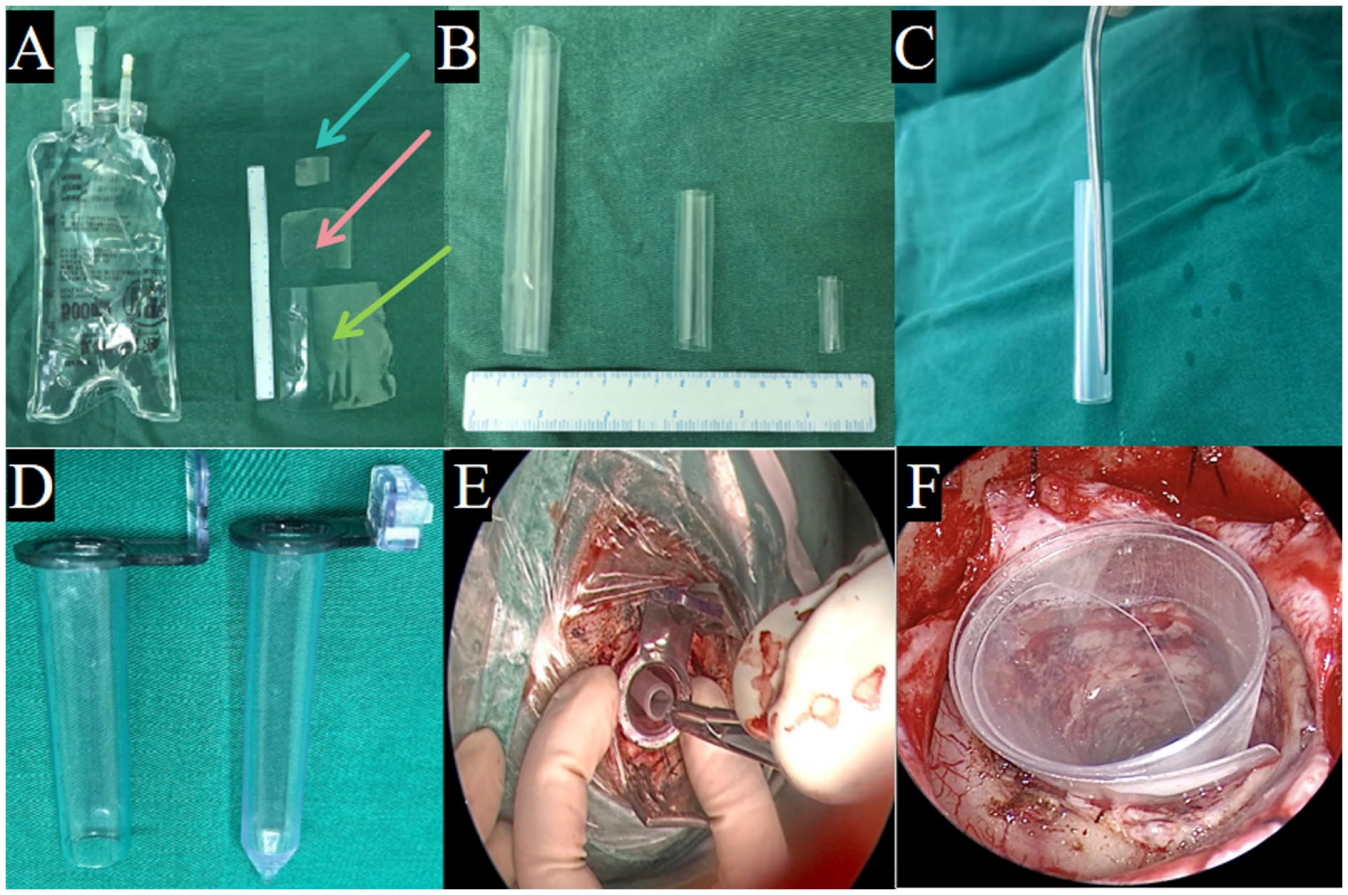
Fabrication and insertion of sheath for IVH evacuation. **(A)** TSNCT sheaths crafted from biocompatible polyvinyl chloride (sterile saline bags), cut into outer sheath (→ green), middle sheath (→ pink), and mini sheath (→ blue). **(B)** Sheaths are rolled into compact sleeves. **(C)** Sleeves secured with gun-shaped tweezers. **(D)** A disposable rigid endoport used to create a surgical channel. **(E)** The sheath inserted through the endoport into the hematoma cavity. **(F)** The rigid endoport is removed, allowing the sheath to expand into a tubular channel.

The TSNCT was performed by neurosurgeons with at least 5 years of neuroendoscopic experience, under the supervision of a senior neurosurgeon with over 10 years of expertise. The technique utilized three transparent sheaths crafted from medical-grade polyvinyl chloride (sterile saline bags) for biocompatibility and flexibility. Sheath fabrication involved:

Cutting saline bags into strips (6 × 6 cm for outer sheath, 4 × 4 cm for middle sheath, 2 × 2 cm for mini sheath) under sterile conditions in a laminar flow hood (Figure 2A). Rolling strips into compact sleeves (Figure 2B), secured with gun-shaped tweezers (Figure 2C).

Inserting the sleeve through a disposable rigid endoport (10 mm diameter) to create a surgical channel (Figures 2D,E), then removing the endoport to allow sheath expansion within the ventricle (Figure 2F).

The sheaths were deployed as follows:

Outer sheath: 8–16 mm diameter, inserted via a frontal burr hole (Kocher’s point: 10–12 cm from nasion, 2–3 cm lateral to midline) to a depth of 4–6 cm to access the lateral ventricle.
Middle sheath: 6–8 mm diameter, advanced through the foramen of Monro (angled 10–15° posteriorly) to the third ventricle, protecting adjacent structures (e.g., fornix, choroid plexus).
Mini sheath: 2 mm diameter, navigated through the midbrain aqueduct (2–4 mm diameter) to a depth of 1–2 cm beyond the aqueduct to access the fourth ventricle, with gentle advancement to avoid trauma.

Hematoma evacuation was performed sequentially from the lateral, third, and fourth ventricles under direct visualization using a 4-mm rigid endoscope (Karl Storz Hopkins II, 0° and 30° lenses, 1,920 × 1,080 resolution, Xenon 300 W light source). Irrigation used warm saline (37°C, 5–10 mL/min via syringe pump) to maintain visibility, with intermittent suction (50–100 mmHg). Hemostasis was achieved with bipolar coagulation. Mini sheath advancement was guided by real-time endoscopic visualization; if significant bleeding occurred, the procedure was paused for hemostasis. Cerebrospinal fluid (CSF) flow was monitored post-evacuation to ensure ventricular patency.

#### Single-port neuroendoscopy group (*n* = 2)

Single-port neuroendoscopy was performed using a 6-mm rigid endoscope (Karl Storz Hopkins II, 0° and 30° lenses, 1,920 × 1,080 resolution) inserted through a frontal burr hole (Kocher’s point) to a depth of 4–6 cm. Hematomas were evacuated from the lateral and third ventricles using suction (50–100 mmHg) and irrigation (warm saline, 5–10 mL/min). Access to the fourth ventricle was limited by the fixed sheath diameter (6 mm) and inability to navigate the midbrain aqueduct. Urokinase (10,000–20,000 IU) was administered postoperatively in select cases (e.g., Case 3) to address residual clots. Intraoperative hemostasis followed the same protocol as TSNCT.

#### EVD group (*n* = 1)

Standard EVD was performed to relieve ICP using a 3-mm Codman EDS 3 catheter (Johnson & Johnson) inserted via a frontal burr hole (Kocher’s point) to a depth of 5–7 cm, targeting the frontal horn of the lateral ventricle. The catheter was connected to a closed drainage system, with CSF drainage set at 10–15 cm H₂O above the external auditory meatus. ICP was monitored continuously (target <20 mmHg), and saline irrigation or urokinase (10,000–20,000 IU, if indicated) was administered per institutional protocols to facilitate clot lysis. Catheter placement was confirmed via postoperative CT.

### Outcome measures

#### Hematoma volume

Measured pre-and postoperatively using 3D Slicer software (version 4.11) via manual segmentation of CT images (1 mm slice thickness, 512 × 512 matrix). Clearance rate was calculated as [(preoperative volume − postoperative volume)/preoperative volume] × 100%.

#### Neurological status

Assessed using the GCS at admission and discharge by trained neurosurgeons, with intubated patients denoted by “T” (verbal score unassessable).

#### Complications

Monitored daily for postoperative infections (e.g., meningitis, pneumonia), rebleeding, persistent hydrocephalus, or mortality via clinical exams and CT scans.

#### Follow-up

Recorded discharge status and planned post-hospital care.

#### Data analysis

Descriptive statistics (means, percentages) were used to summarize patient characteristics (age, gender), hematoma clearance rates, GCS changes, operation times, hospital stay durations, and complication rates. A comparative analysis of outcomes (clearance rate, neurological improvement, complications, mortality) was conducted between TSNCT (Cases 1–2), standard endoscopic hematoma removal (Cases 3–4), and EVD (Case 5). No inferential statistical tests were applied due to the small sample size (*n* = 5), consistent with the pilot study design. Data were analyzed using Microsoft Excel (version 16.78).

## Results

### Illustrative cases

Cases are presented in a standardized format: clinical presentation, surgical procedure, treatment outcomes, and follow-up plans.

### Case 1

#### Clinical presentation

**Figure 3:**
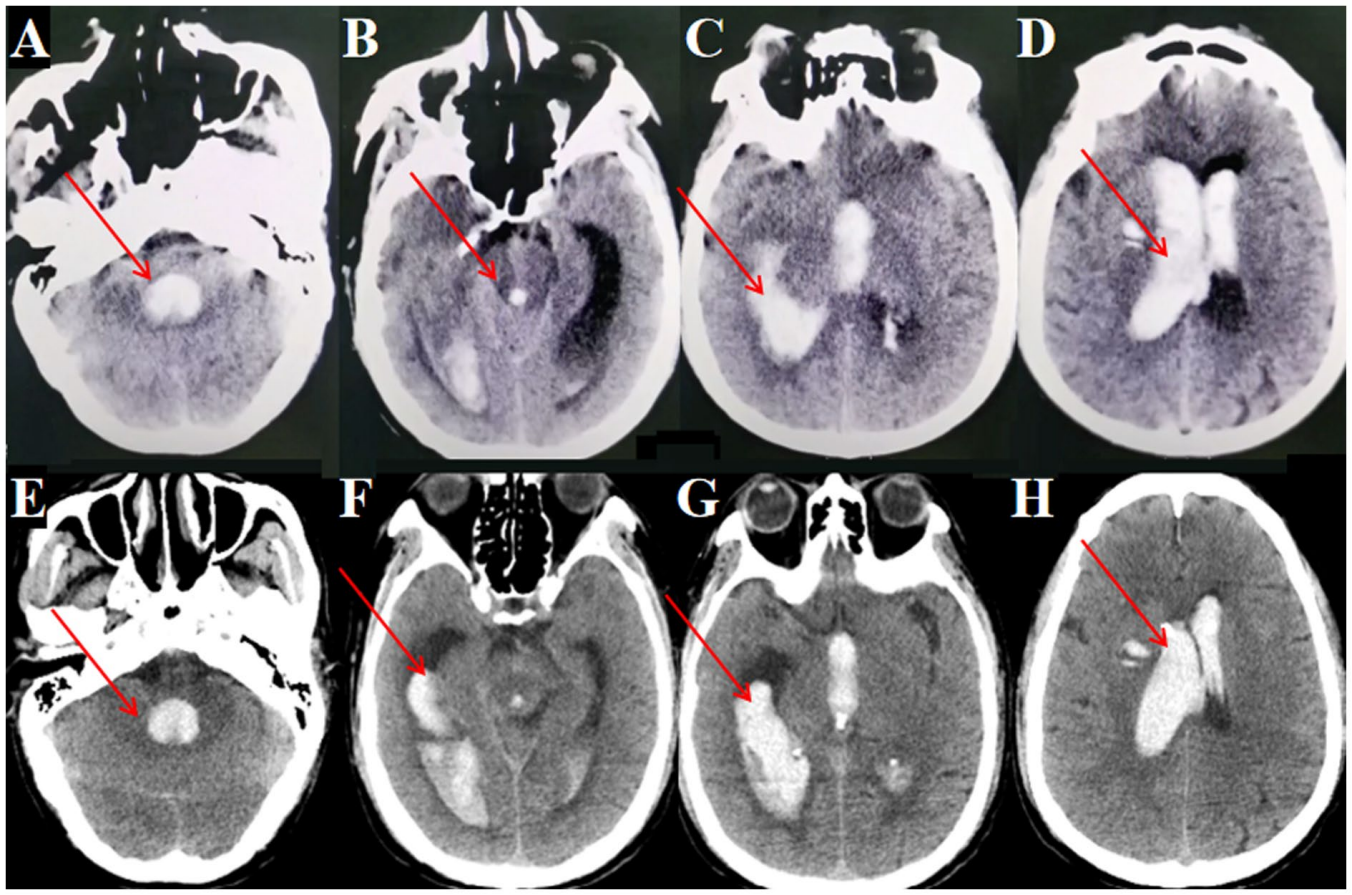
Preoperative and postoperative cranial CT imaging. **(A, B)** Cranial CT showing fourth ventricular hemorrhage casting (→ arrows indicate blood in the fourth ventricle). **(C)** Cranial CT showing lateral ventricular hemorrhage casting (→ arrow marks clot in the right lateral ventricle). **(D)** Cranial CT showing third ventricular hemorrhage casting (→ arrows indicate hematoma in the lateral and third ventricles). **(E, F)** Postoperative cranial CT after bilateral EVD (performed at local hospital) showing persistent fourth ventricular hemorrhage casting (→ arrows highlight residual clot in the fourth ventricle). **(G, H)** Postoperative CT after bilateral EVD showing persistent lateral and third ventricular hemorrhage casting (→ arrows mark residual hematoma in the lateral and third ventricles).

A 61-year-old male was admitted after experiencing sudden dizziness, nausea, vomiting, and limb weakness about 7 h before admission. His condition worsened, leading to altered consciousness. A cranial CT scan revealed hemorrhages in the bilateral lateral ventricles, third ventricle, and fourth ventricle, along with complete ventricular casting and hydrocephalus (Figures 3A–D, arrows indicate hematoma in all ventricles). An emergency ventricular puncture and drainage were performed at a local hospital to relieve ICP, followed by transfer to our facility. A follow-up CT scan showed persistent hemorrhage and hydrocephalus (Figures 3E–H).

#### Surgical procedure

**Figure 4:**
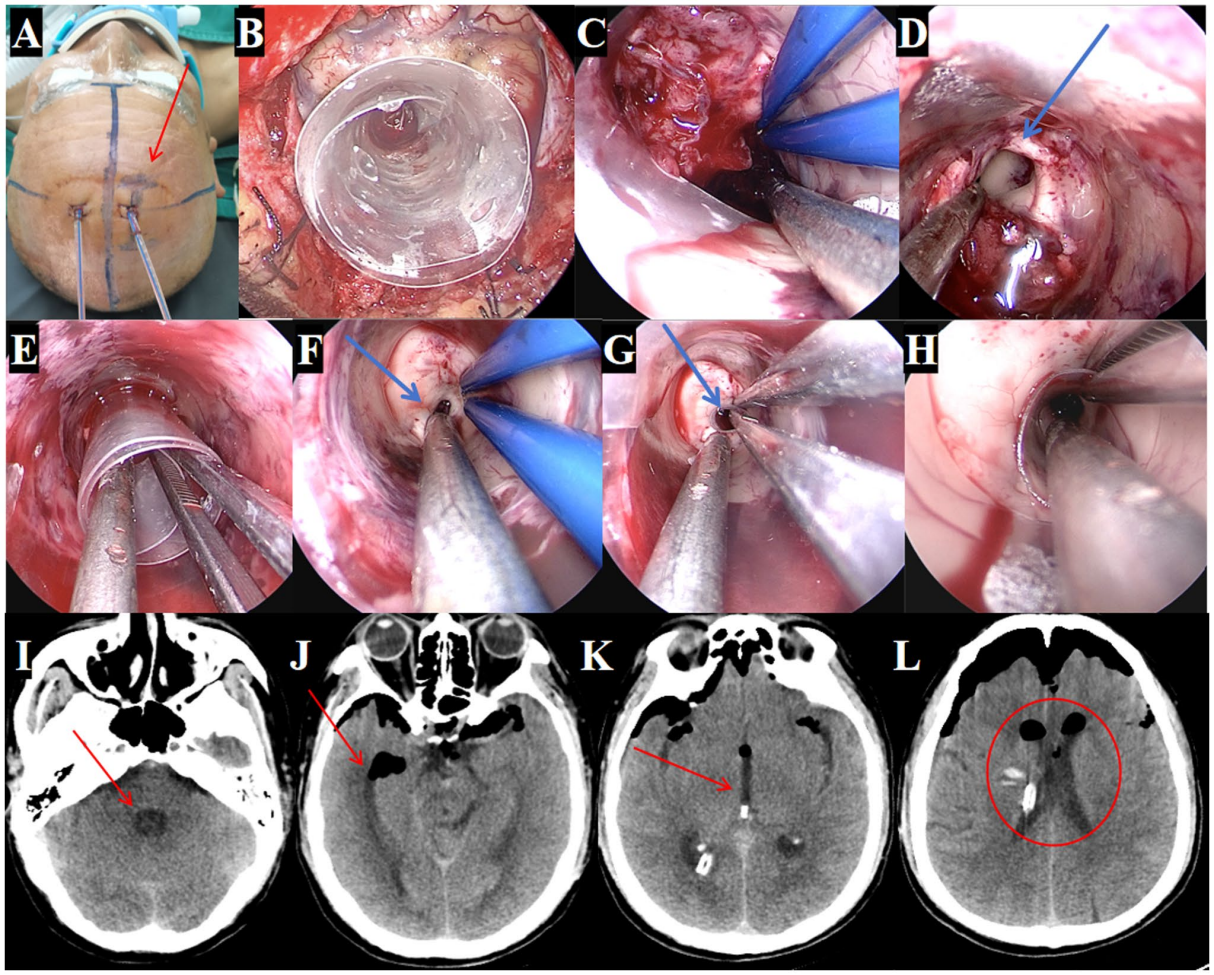
Surgical procedure using the TSNCT for treating patients with complete ventricular casting. **(A)** Selection of the existing right lateral ventricle frontal horn approach (→ arrow marks entry site). **(B)** Insertion of a outer sheath through the puncture pathway. **(C)** Removal of hematoma from the lateral ventricle. **(D)** Clearance of hematoma in the foramen of Monro region (→ arrow highlights cleared foramen). **(E)** Insertion of the middle sheath through the foramen of Monro. **(F, G)** Exploration of the midbrain aqueduct (→ arrow marks aqueduct lumen). **(H)** Insertion of a mini-sheath through the midbrain aqueduct and removal of hematoma from the fourth ventricle via the mini-sheath. **(I–L)**. Postoperative CT indicating successful hematoma clearance (→ arrows mark evacuated regions; ◯ circles show restored ventricular patency).

Despite the initial drainage, the hematoma did not show significant improvement. The decision was made to perform an endoscopic hematoma removal. A linear incision of approximately 3 cm was marked at the right lateral ventricle’s frontal horn drainage site (Figure 4A). During surgery, a TSNCT was employed, using an outer sheath inserted into the right lateral ventricle, a middle sheath placed through the foramen of Monro for third ventricle access, and a mini sheath inserted into the midbrain aqueduct to remove hematoma from the fourth ventricle (Figures 4B-H). Thorough hemostasis was achieved, and sheaths were withdrawn.

#### Treatment outcomes

**Figure 5:**
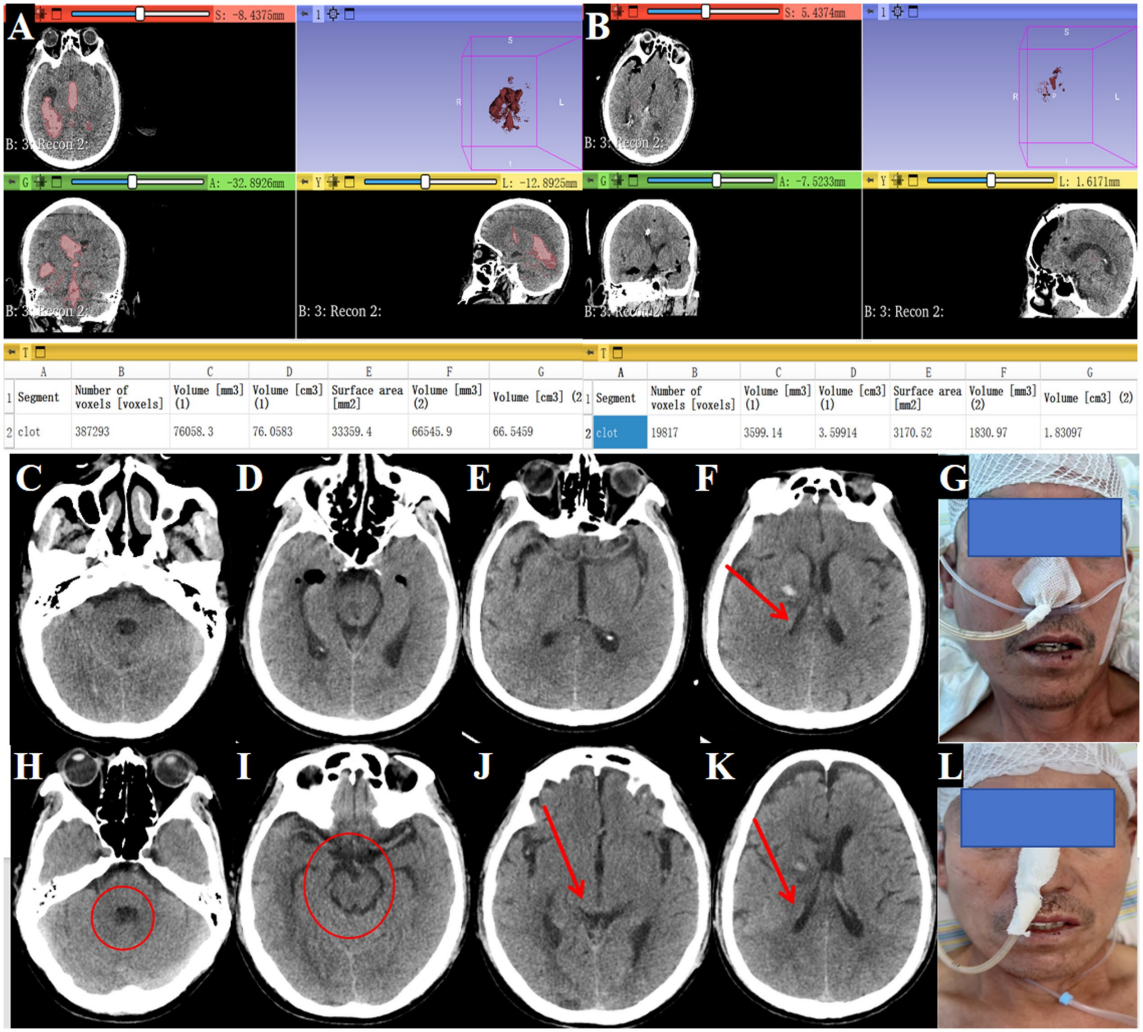
Postoperative improvement in consciousness and outcomes. **(A)** Preoperative head CT showing hematoma volume of 76.1 mL (→ arrows mark hemorrhage in lateral, third, and fourth ventricles). **(B)** Postoperative head CT showing hematoma volume reduced to 3.6 mL (→ arrows indicate evacuated regions; ◯ circles highlight restored ventricular patency). **(C–F)** Drainage tube removal on postoperative day 3 (→ arrow marks tube site). **(G)** Patient with improved consciousness responsiveness shown in clinical image. **(H–K)** Tenth postoperative day CT showing no hydrocephalus (→ arrows denote normalized ventricular size; ◯ circles confirm absence of residual clots). **(L)** The patient was alert and neurologically stable.

Postoperative CT confirmed near-complete hematoma evacuation from all affected ventricles (Figures 4I–L). Postoperative CT confirmed 95.3% hematoma clearance (preoperative volume: 76.1 ml, postoperative: 3.6 ml; Figures 5A,B) calculated by 3D Slicer software. GCS improved from 4T to 14. The EVD tube was removed on postoperative day 3, (Figures 5C–G) patient showing improved consciousness and no signs of hydrocephalus by day 10 (Figures 5H–L).

#### Follow-up plans

Discharged after 17 days; referred to outpatient rehabilitation.

### Case 2

#### Clinical presentation

**Figure 6:**
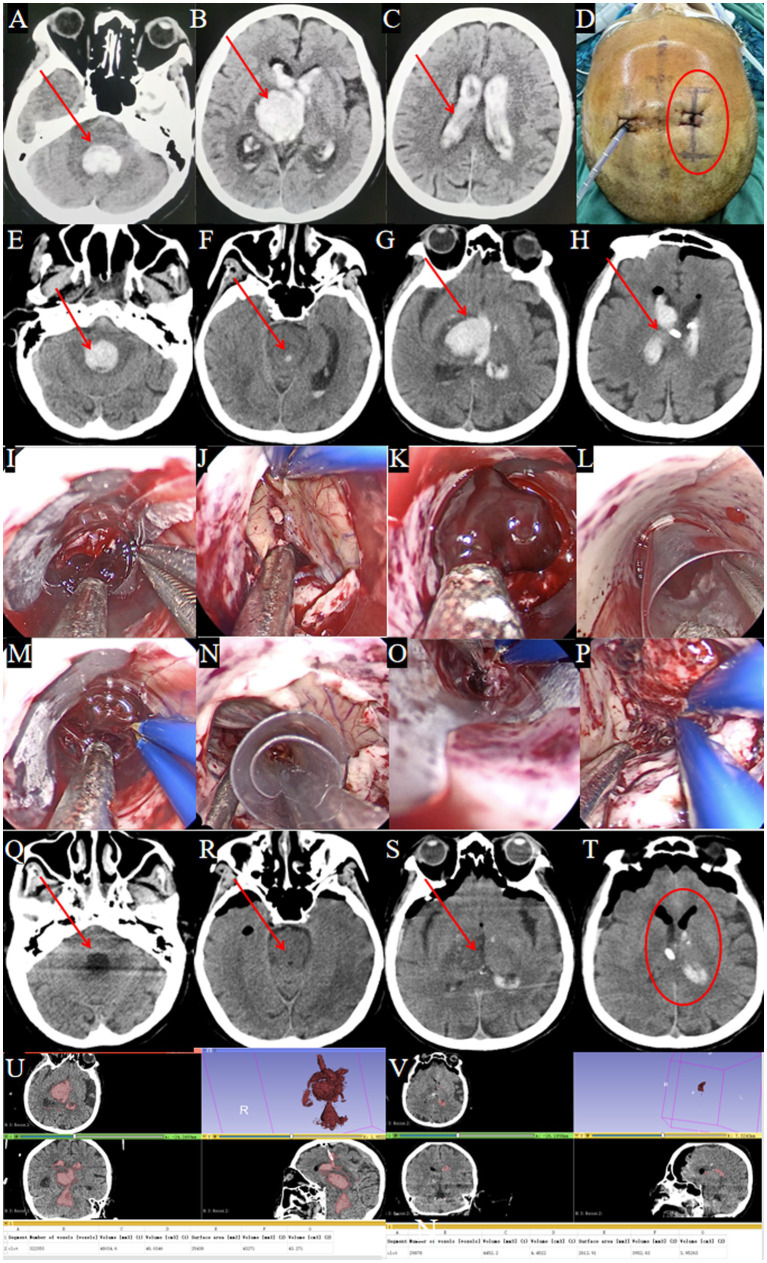
Surgical procedure using the TSNCT for complete ventricular casting. **(A–D)** Patient transferred after EVD at a local hospital (→ arrows in **A–C** show residual clots in fourth, third and lateral ventricles; ◯ circles in **D** mark the right frontal horn approach site). **(E–H)** Admission CT showing complete ventricular casting (→ arrows indicate hemorrhage in all ventricles). **(I)** Insertion of the outer sheath through the puncture pathway. **(J)** Hematoma removal from the lateral ventricle. **(K)** Clearance of hematoma at the foramen of Monro. **(L)** Insertion of the middle sheath into the third ventricle. **(M)** Exploration of the midbrain aqueduct. **(N)** Insertion of the mini-sheath through the midbrain aqueduct. **(O)** Hematoma removal from the fourth ventricle. **(P)** Endoscopic view confirming complete evacuation of the fourth ventricle. **(Q–T)** Postoperative CT showing successful hematoma clearance (→ arrows denote evacuated ventricles; ◯ circles confirm restored anatomy). **(U)** Preoperative CT: hematoma volume = 48 mL. **(V)** Postoperative CT: hematoma volume = 4.5 mL.

A 70-year-old female was admitted after experiencing sudden altered consciousness about 5 h prior to arrival. A cranial CT scan at a local hospital revealed thalamic hemorrhage rupturing into the ventricular system, causing bilateral lateral ventricle, third ventricle, and fourth ventricle casting, along with hydrocephalus (Figures 6A–C, arrows mark hematoma). Emergency ventricular puncture and drainage were performed (Figure 6D). Upon transfer to our hospital, a follow-up CT scan showed persistent hemorrhage (Figures 6E–H).

#### Surgical procedure

TSNCT was performed using the existing right lateral ventricle drainage site. An outer sheath accessed the lateral ventricle, a middle sheath cleared the third ventricle via the foramen of Monro, and a mini sheath navigated the midbrain aqueduct to evacuate the fourth ventricle hematoma (Figures 6I–P). Cerebrospinal fluid circulation was restored, and hemostasis was confirmed.

#### Treatment outcomes

Treatment Outcomes: Postoperative CT confirmed near-complete removal of the hematomas from all affected ventricles (Figures 6Q–T). Postoperative CT showed 90.6% hematoma clearance (preoperative volume: 48.0 ml, postoperative: 4.5 ml; Figures 6U,V). GCS improved from 4T to 8T. A temporary tracheostomy was required due to pulmonary infection.

### Follow-up plans

Hospitalized for 17 days; discharged to a rehabilitation facility.

### Case 3

#### Clinical presentation

**Figure 7:**
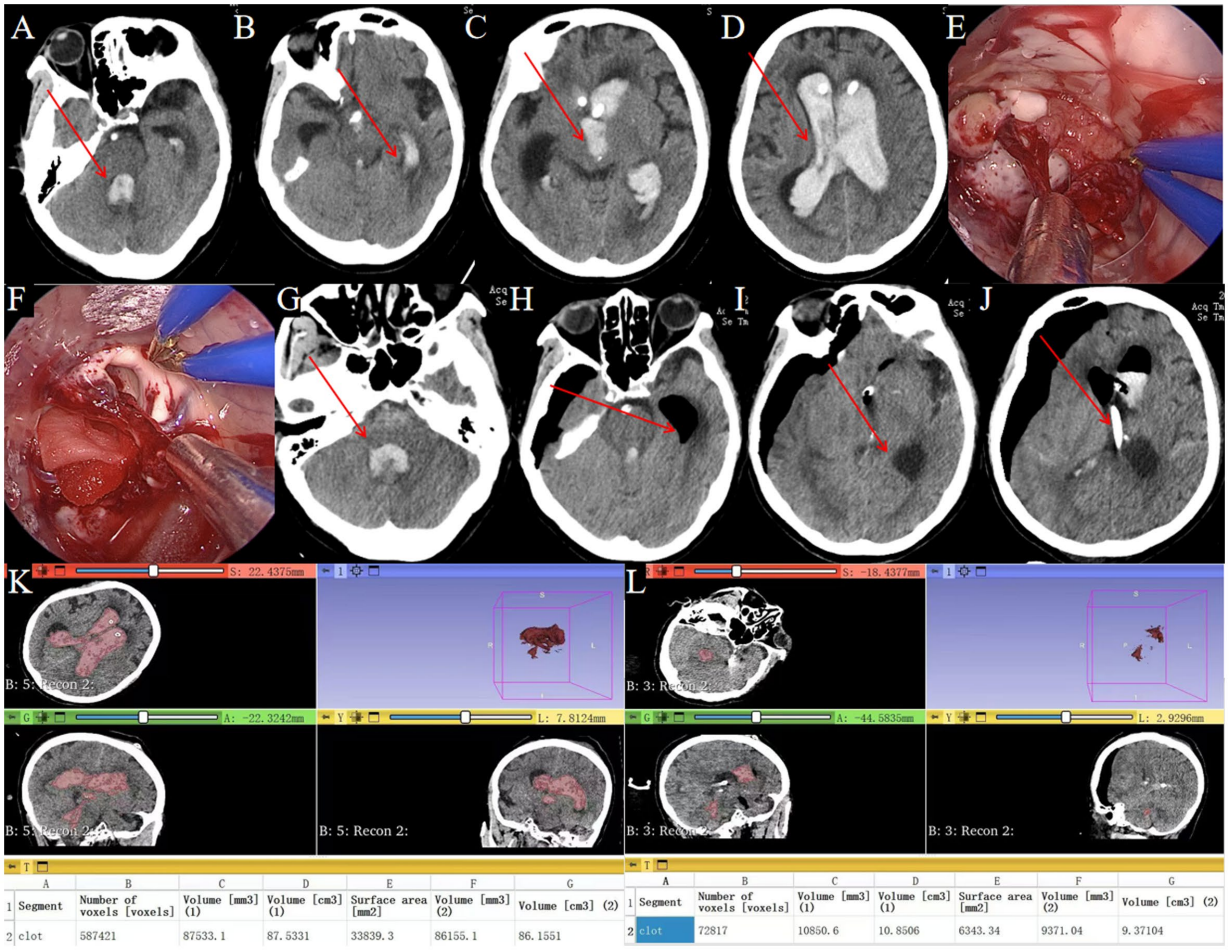
Endoscopic removal of hematoma in complete ventricular casting. **(A–D)** Preoperative head CT showing complete ventricular casting (→ arrows mark hemorrhage in the fourth, third and lateral ventricles). **(E)** Intraoperative neuroendoscopic view of hematoma evacuation from the lateral ventricle using a transparent sheath. **(F)** Intraoperative neuroendoscopic view confirming clearance of the lateral ventricle. **(G)** Postoperative CT showing residual hematoma in the fourth ventricle (→ arrow highlights residual clot). **(H,I)** Postoperative CT demonstrating hematoma removal in the lateral and third ventricles (→ arrows denote evacuated regions). **(J)** Postoperative CT showing drainage tube to drain residual hematoma (→ arrows denote drainage tube). **(K)** Preoperative hematoma volume: 87.5 mL. **(L)** Postoperative hematoma volume: 10.8 mL.

A 66-year-old male was admitted after experiencing altered consciousness for 2 days. He had undergone bilateral ventricular puncture and drainage at another hospital, but consciousness did not improve significantly. A follow-up cranial CT scan upon admission revealed ventricular casting in the bilateral lateral ventricles, third ventricle, and fourth ventricle, along with hydrocephalus (Figures 7A–D, arrows indicate hematoma).

#### Surgical procedure

Standard endoscopic hematoma removal was performed, targeting the lateral and third ventricles (Figures 7E–F). The absence of the three-sheath system limited access to the midbrain aqueduct and fourth ventricle. Urokinase was administered via the drainage tube post-surgery.

#### Treatment outcomes

Postoperative CT showed residual hematomas in the fourth ventricle (Figure 7G) and successful evacuation of lateral/third ventricles (Figures 7H–I). A drainage tube was placed for residual clots (Figure 7J). Postoperative CT showed 87.6% hematoma clearance (preoperative volume: 87.5 mL, postoperative: 10.8 mL; Figures 7K,L). GCS improved from 4T to 8T. A tracheostomy was required due to pulmonary infection.

#### Follow-up plans

Discharged after 39 days, able to follow simple commands; transferred to rehabilitation.

### Case 4

#### Clinical presentation

**Figure 8:**
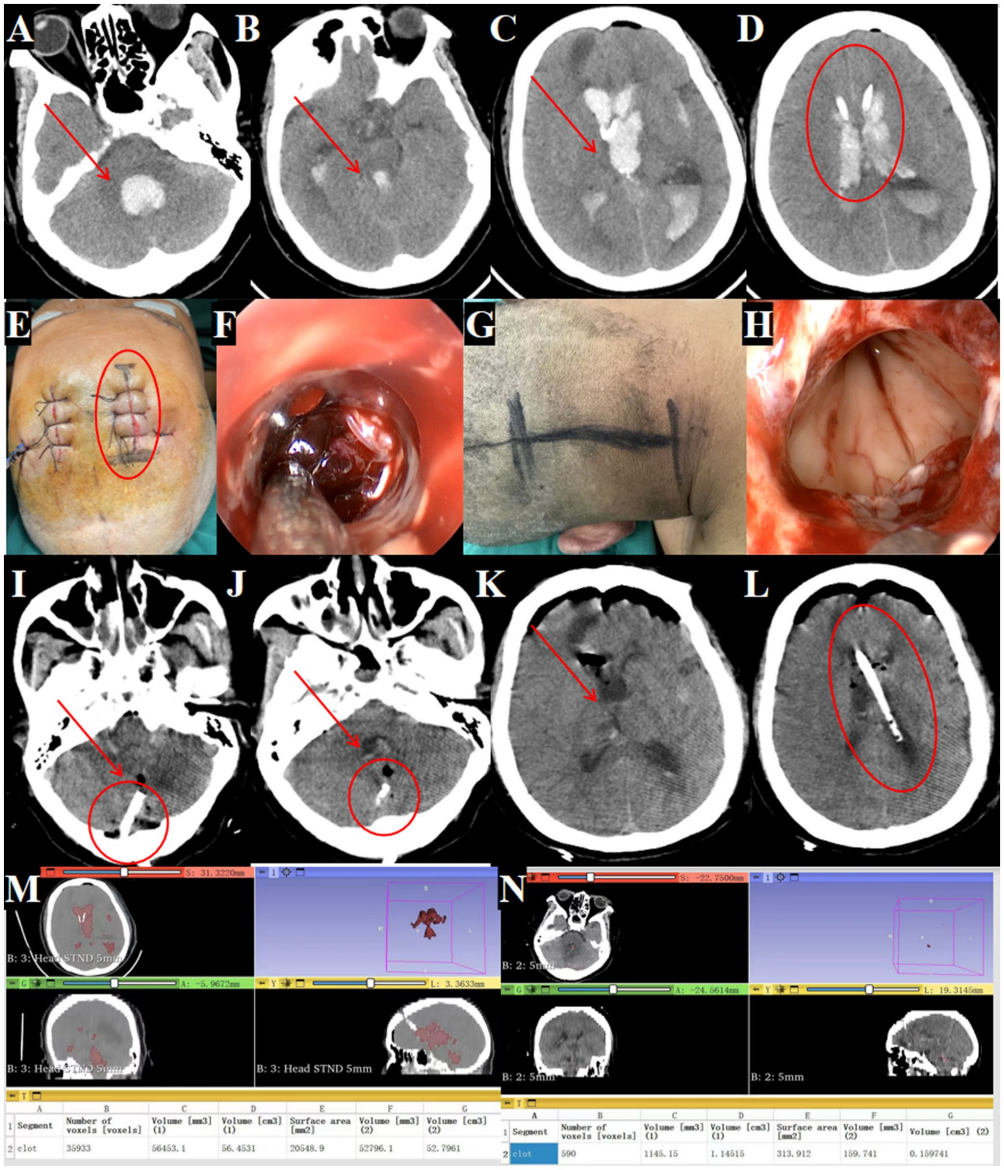
Management of fourth ventricular hematoma requiring dual surgical approaches. **(A–D)** Post-transfer cranial CT after EVD at a local hospital (→ arrows in **A–C** show residual hemorrhage in the fourth, third and lateral ventricles; ◯ circle in **D** marks the external drainage site). **(E,F)** Supratentorial approach for hematoma removal (◯ circle indicates entry point). **(F)** Evacuation of hematoma in the lateral and third ventricles. **(G)** Infratentorial approach for fourth ventricular hematoma removal. **(H)** Evacuation of hematoma in the fourth ventricle. **(I–L)** Postoperative CT confirming complete hematoma clearance (→ arrows denote evacuated ventricles; ◯ circles show drainage tubes). **(M)** Preoperative hematoma volume: 56.4 mL. **(N)** Postoperative hematoma volume: 1.1 mL.

A 57-year-old female was admitted after sudden loss of consciousness lasting 1 day. She had undergone bilateral ventricular puncture and drainage at another hospital without significant improvement. A cranial CT scan showed ventricular casting in both lateral ventricles, the third ventricle, and the fourth ventricle, along with hydrocephalus (Figures 8A–D, arrows mark hematoma).

#### Surgical intervention

Standard endoscopic removal was performed via a right frontal approach for the lateral and third ventricles (Figures 8E–F) and a posterior midline approach for the fourth ventricle, requiring two incisions (Figures 8G,H).

#### Treatment outcomes

Post-operative CT confirmed near complete removal of the hematomas from all affected ventricles (Figures 8I,L) with 98.1% hematoma clearance (preoperative volume: 56.4 ml, postoperative: 1.1 ml; Figures 8M,N). GCS improved from 5T to 7T. A tracheostomy was needed due to pulmonary infection.

#### Follow-up plans

Discharged after 39 days with vague consciousness; transferred to rehabilitation.

### Case 5

#### Clinical presentation

**Figure 9:**
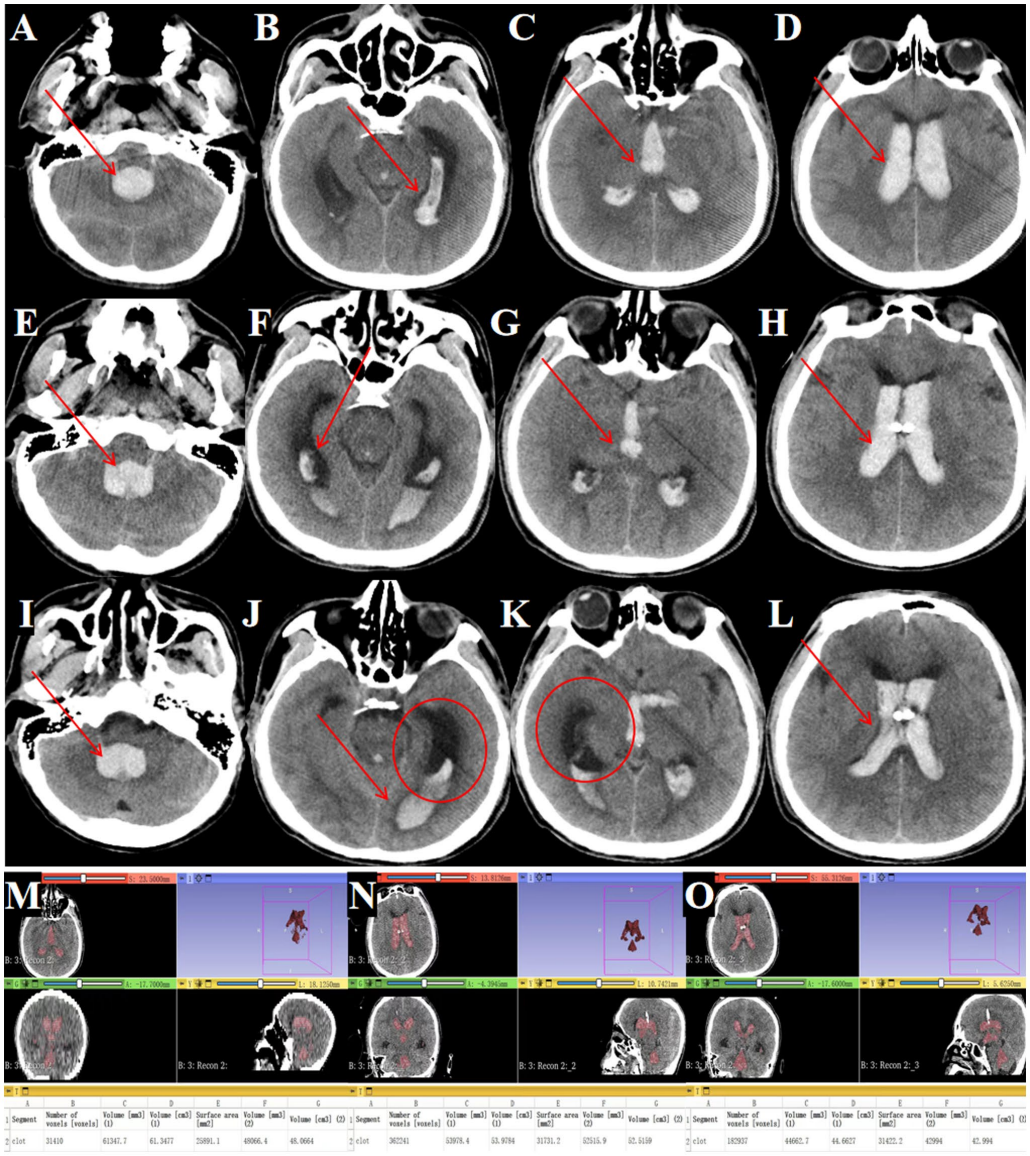
Outcomes of traditional EVD in complete IVH casting. **(A–D)** Admission head CT showing complete IVH casting (→ arrows mark hemorrhage in the fourth, third and lateral ventricles). **(E–H)** Postoperative CT after bilateral EVD (→ arrows highlight residual hematoma in all ventricles). **(I–L)** Day 3 post-EVD CT demonstrating ongoing IVH casting (→ arrows denote unresolved hemorrhage; ◯ circles confirm worsening ventricular dilation). **(M)** Preoperative hematoma volume: 61.3 mL. **(N)** Postoperative hematoma volume reduced to 53.9 mL. **(O)** Day 3 post-surgery hematoma volume: 44.6 mL.

A 52-year-old male was admitted with a sudden headache and consciousness disturbance lasting over 2 h. A head CT at another hospital revealed complete IVH casting, prompting transfer for emergency bilateral intraventricular drainage. Upon admission, the patient was in a deep coma, unresponsive to painful stimuli, with pupils measuring 2.5 mm and no light reflex, neck rigidity, and poor oxygen saturation (Figures 9A–D, arrows indicate hematoma).

#### Surgical procedure

Bilateral EVD was performed due to the patient’s critical condition and poor prognosis.

#### Treatment outcomes

Serial imaging demonstrated limited hematoma resolution, with residual intraventricular blood seen immediately post-EVD (Figures 9E–H) and persistent casting at day 3 (Figures 9I–L). Quantitative analysis showed only 27.2% hematoma clearance (preoperative volume: 61.3 ml; Figure 9M, postoperative: 53.9 ml; Figure 9N, day 3: 44.6 ml; Figure 9O).

#### Follow-up plans

None (deceased).

### Comparative outcomes of TSNCT versus traditional approaches

A comparative analysis of outcomes between TSNCT (Cases 1 and 2), standard endoscopic hematoma removal (Cases 3 and 4), and external ventricular drainage (EVD, Case 5) was conducted based on hematoma clearance, neurological improvement, complications, and mortality.

#### Hematoma clearance rate

TSNCT achieved a mean clearance rate of 93.5% (preoperative volume: 62.05 mL, postoperative: 4.05 mL), compared to 91.7% for standard endoscopic removal (preoperative: 71.95 mL, postoperative: 5.95 mL) and 27.2% for EVD (preoperative: 61.3 mL, postoperative: 44.6 mL). These data suggest TSNCT may enhance hematoma evacuation, particularly in complex IVH cases.

#### Neurological improvement

TSNCT patients showed a mean Glasgow Coma Scale (GCS) improvement of 7 points at discharge (Case 1: 4 T to 14; Case 2: 4 T to 8 T), versus 3 points for standard endoscopic removal (Case 3: 4 T to 8 T; Case 4: 5 T to 7 T) and no improvement for EVD (Case 5: 3 to 3), indicating potential for greater neurological recovery with TSNCT.

#### Complication rates

Complications occurred in 50% of TSNCT cases (1/2, pulmonary infection in Case 2), 100% of standard endoscopic cases (2/2, pulmonary infections in Cases 3 and 4), and 0% of EVD cases (though Case 5 expired). The small sample size limits firm conclusions.

#### Mortality

Mortality was 0% for TSNCT and standard endoscopic removal, but 100% for EVD (Case 5), reflecting the patient’s severe condition.

## Discussion

Patients with ventricular casting, especially those with fourth ventricular involvement due to ruptured aneurysms, present in critical condition, where mortality rates can reach up to 93% (14). Recent studies using advanced neuroendoscopic sheath-assisted techniques, such as the heron-mouth design (15), have demonstrated improved hematoma clearance (90.6%) and reduced complications compared to traditional EVD. However, severe hemorrhage affecting all ventricular chambers remains associated with high mortality (16). The management of complete IVH casting thus presents significant clinical challenges, emphasizing the urgent need for innovative treatment strategies to enhance patient survival and recovery.

The TSNCT emerges as a pivotal advancement in addressing these challenges. This method uniquely tackles the complexities involved in hematoma evacuation from anatomically constrained regions, providing a nuanced approach to a condition traditionally difficult to treat. Recent literature underscores the efficacy of neuroendoscopic interventions, especially with flexible endoscopes, in markedly reducing ICP and improving outcomes by evacuating blood clots from the ventricles (17, 18). However, the midbrain aqueduct’s narrow diameter of approximately 2 mm has historically restricted access to the fourth ventricle, resulting in suboptimal outcomes for patients with complete IVH casting.

Alternative approaches, such as the cerebellar medulla fissure technique described by Gao et al. (19), have shown success in managing severe ventricular hemorrhage but do not directly address the challenge of navigating the midbrain aqueduct. In contrast, TSNCT, pioneered by Professor Cai Qiang at Renmin Hospital of Wuhan University, is specifically designed to surmount this limitation. The mini sheath enables precise navigation through the aqueduct, facilitating comprehensive hematoma evacuation across all ventricular chambers under direct neuroendoscopic visualization. This study’s preliminary results, with a mean hematoma clearance rate of 93.5% in TSNCT cases, suggest that the technique achieves more thorough clot removal compared to traditional methods, potentially improving clinical outcomes.

The cases presented illustrate TSNCT’s practical advantages. Cases 1 and 2, treated with TSNCT, achieved clearance rates of 95.3 and 90.6%, respectively, with a mean GCS improvement of 7 points, reflecting substantial neurological recovery. In contrast, Cases 3 and 4, managed with standard endoscopic hematoma removal, achieved a mean clearance rate of 91.7% but only a 3-point GCS improvement, likely due to incomplete fourth ventricle evacuation. The need for dual incisions in Case 4 further highlights the increased procedural complexity and risk associated with traditional methods. Case 5, treated solely with EVD, underscores the inadequacy of non-surgical approaches, with only 27.2% hematoma clearance, no GCS improvement, and patient mortality following withdrawal of care. This comparison highlights TSNCT’s potential to evacuate deep-seated hematomas, particularly in the fourth ventricle—a region inaccessible to EVD and standard endoscopy.

TSNCT’s benefits extend beyond hematoma clearance. By minimizing the need for multiple surgical entries, as seen in Case 4, the technique reduces procedural risks and iatrogenic damage. Preliminary data show complications in one of two TSNCT cases (50%) compared to both standard endoscopy cases (100%), though the small sample size limits conclusions. Additionally, TSNCT’s comprehensive evacuation may shorten hospital stays and reduce rehabilitation demands, offering potential socioeconomic benefits that warrant further exploration.

The integration of TSNCT into the CODE ICH (comprehensive optimization of determinants in intracerebral hemorrhage) framework, a stage-based approach to intracerebral hemorrhage management (20), enhances its clinical relevance. In the acute phase, where rapid hematoma evacuation and ICP reduction are critical, TSNCT’s high clearance rates and minimally invasive nature position it as a valuable option for high-risk IVH patients. Unlike EVD, which provides only temporary ICP relief (as in Case 5), or standard endoscopy, which struggles with fourth ventricle access, TSNCT offers a targeted solution that aligns with CODE ICH’s emphasis on tailored interventions. Future studies should explore specific indications for TSNCT within this framework to optimize patient selection and timing.

Compared to other emerging techniques, such as stereotactic aspiration or flexible neuroendoscopy without sheath systems, TSNCT’s structured multi-sheath approach provides superior access and precision. However, its technical complexity requires specialized training, which may limit immediate adoption. Ongoing advancements in endoscopic technology, such as improved sheath materials or enhanced visualization systems, could further refine TSNCT’s efficacy and accessibility.

The comparative outcomes presented in the Results are exploratory due to the small sample size (*n* = 5) and pilot nature of this study. Larger, prospective studies are needed to validate these findings.

### Limitations

This pilot observational study has several limitations. The small sample size (*n* = 5), with only two TSNCT cases, restricts statistical power and generalizability. The retrospective design and lack of randomization introduce selection bias, as treatment decisions were based on clinical judgment and changes in institutional practices over time. The absence of a matched control group precludes definitive claims about TSNCT’s superiority. Additionally, the short-term follow-up (up to discharge) limits insights into long-term outcomes, such as functional recovery or quality of life. Finally, TSNCT’s technical demands may lead to variability in outcomes depending on surgical expertise, a factor not fully assessed. Prospective, multicenter studies with larger, randomized cohorts and extended follow-up are essential to validate these findings and establish standardized protocols.

### Future directions

Future research should focus on expanding TSNCT’s application across diverse patient populations, refining procedural techniques, and evaluating long-term outcomes. Comparative trials against other minimally invasive methods, such as flexible neuroendoscopy or stereotactic aspiration, will clarify TSNCT’s relative efficacy. Additionally, studies assessing cost-effectiveness and training requirements will facilitate broader clinical adoption, ensuring TSNCT’s transformative potential is realized in neurosurgical practice.

## Conclusion

The TSNCT shows promise as an innovative approach for managing complete IVH casting in patients with cerebral hemorrhage. By enabling near-complete hematoma evacuation in anatomically challenging regions, such as the midbrain aqueduct, TSNCT enhances surgical precision and shows the potential to improve neurological outcomes, as evidenced by higher clearance rates and greater GCS improvements compared to traditional methods in this pilot study. However, the small sample size and preliminary nature of these findings necessitate further prospective, multicenter studies to validate TSNCT’s efficacy, establish standardized protocols, and assess long-term outcomes. Continued research will be critical to realizing TSNCT’s potential as a transformative tool in neurosurgical practice for severe IVH management.

## Data Availability

The datasets presented in this article are not readily available because of ethical and privacy restrictions. Requests to access the datasets should be directed to the corresponding authors.

## References

[1] Zada G Liu C Apuzzo ML. “Through the looking glass”: optical physics, issues, and the evolution of neuroendoscopy. World Neurosurg. (2013) 79:S3–S13. doi: 10.1016/j.wneu.2013.02.001 23391453

[2] Di Ieva A Tam M Tschabitscher M Cusimano MD. A journey into the technical evolution of neuroendoscopy. World Neurosurg. (2014) 82:e777–89. doi: 10.1016/j.wneu.2014.09.005 25225133

[3] Grunert P Gaab MR Hellwig D Oertel JM. German neuroendoscopy above the skull base. Neurosurg Focus. (2009) 27:E7. doi: 10.3171/2009.6.FOCUS09123 19722822

[4] Shim KW Park EK Kim DS Choi JU. Neuroendoscopy: current and future perspectives. J Korean Neurosurg Soc. (2017) 60:322–6. doi: 10.3340/jkns.2017.0202.006 28490159 PMC5426450

[5] Hinson HE Hanley DF Ziai WC. Management of intraventricular hemorrhage. Curr Neurol Neurosci Rep. (2010) 10:73–82. doi: 10.1007/s11910-010-0086-6 20425231 PMC3138489

[6] Godoy Hurtado A Barstchi P Brea Salvago JF Al-Ghanem R Galicia Bulnes JM El Rubaidi O. Low-and negative-pressure hydrocephalus: new report of six cases and literature review. J Clin Med. (2023) 12:4112. doi: 10.3390/jcm12124112, PMID: 37373809 PMC10299038

[7] Haldrup M Rasmussen M Mohamad N Dyrskog S Thorup L Mikic N. Intraventricular lavage vs. external ventricular drainage for intraventricular hemorrhage: a randomized clinical trial. JAMA Netw Open. (2023) 6:e2335247:e2335247. doi: 10.1001/jamanetworkopen.2023.35247, PMID: 37815832 PMC10565600

[8] Mezzacappa FM Weisbrod LJ Schmidt CM Surdell D. Neuroendoscopic evacuation improves outcomes compared with external ventricular drainage in patients with spontaneous intraventricular hemorrhage: a systematic review with meta-analyses. World Neurosurg. (2023) 175:e247–53. doi: 10.1016/j.wneu.2023.03.061 36958716

[9] Liu X Qiu Y Zhang F Wei X Zhou Z Zhang F. Combined intra-and extra-endoscopic techniques for endoscopic intraventricular surgery with a new mini-tubular port. Front Surg. (2022) 9:933726. doi: 10.3389/fsurg.2022.933726, PMID: 36081583 PMC9445220

[10] Toyooka T Kageyama H Tsuzuki N Ishihara S Oka K. Flexible endoscopic aspiration for intraventricular casting hematoma. Acta Neurochir Suppl. (2016) 123:17–23. doi: 10.1007/978-3-319-29887-0_3 27637624

[11] Du B Shan AJ Zhang YJ Wang J Peng KW Zhong XL. The intra-neuroendoscopic technique: a new method for rapid removal of acute severe intraventricular hematoma. Neural Regen Res. (2018) 13:999–1006. doi: 10.4103/1673-5374.233442, PMID: 29926826 PMC6022483

[12] Jing S Zhang L Xu L. Analysis of effect of minimally invasive fourth-ventricle hematoma removal for patients with intraventricular hemorrhage casting and influence of feedback early rehabilitation on postoperative neurological function. Altern Ther Health Med. (2024) 30:164–9. 37971461

[13] Di Rienzo A Colasanti R Esposito D Della Costanza M Carrassi E Capece M. Endoscope-assisted microsurgical evacuation versus external ventricular drainage for the treatment of cast intraventricular hemorrhage: results of a comparative series. Neurosurg Rev. (2020) 43:695–708. doi: 10.1007/s10143-019-01110-7, PMID: 31069562

[14] Catapano JS Zabramski JM Baranoski JF Brigeman S Morgan CD Hendricks BK. The prognostic significance of a cast fourth ventricle in ruptured aneurysm patients with intraventricular hemorrhage in the Barrow Ruptured Aneurysm Trial (BRAT). Neurosurgery. (2019) 85:E275–83. doi: 10.1093/neuros/nyy493 30476225

[15] Xu J Ma S Wu W Fang W Zhu A Ge C. Heron-mouth neuroendoscopic sheath-assisted neuroendoscopy plays critical roles in treating hypertensive intraventricular hemorrhage. Wideochir Inne Tech Maloinwazyjne. (2021) 16:199–210. doi: 10.5114/wiitm.2020.99351 33786135 PMC7991947

[16] Essibayi MA Ibrahim Abdallah O Mortezaei A Zaidi SE Vaishnav D Cherian J. Natural history, pathophysiology, and recent management modalities of intraventricular hemorrhage. J Intensive Care Med. (2024) 39:813–9. doi: 10.1177/08850666231204582 37769332

[17] Longatti P Basaldella L. Endoscopic management of intracerebral hemorrhage. World Neurosurg. (2013) 79:S17.e1–7. doi: 10.1016/j.wneu.2012.02.025 22381838

[18] Feletti A Basaldella L Fiorindi A. How I do it: flexible endoscopic aspiration of intraventricular hemorrhage. Acta Neurochir. (2020) 162:3141–6. doi: 10.1007/s00701-020-04499-z 32700081 PMC7593288

[19] Gao F Wang H Wang Z. Clinical application of microsurgery using the cerebellar medulla fissure approach in severe ventricular hemorrhage with casting of the fourth ventricle and its influence on neurological recovery. Evid Based Complement Alternat Med. (2021) 2021:3699233. doi: 10.1155/2021/3699233 34733338 PMC8560247

[20] Li Q Yakhkind A Alexandrov AW Alexandrov AV Anderson CS Dowlatshahi D. Code ICH: a call to action. Stroke. (2024) 55:494–505. doi: 10.1161/STROKEAHA.123.043033, PMID: 38099439

